# Comparison of ^68^Ga-PSMA PET/CT and multiparametric MRI for the detection of low- and intermediate-risk prostate cancer

**DOI:** 10.1186/s13550-022-00881-3

**Published:** 2022-02-11

**Authors:** Chuanchi Zhou, Yongxiang Tang, Zhihe Deng, Jinhui Yang, Ming Zhou, Long Wang, Shuo Hu

**Affiliations:** 1 Department of Urology, The Third Xiangya Hospital, Central South University, 138 Tongzipo Road, Changsha, 410013 Hunan China; 2 Department of Urology, Xiangya Hospital, Central South University, Changsha, Hunan China; 3 Department of Nuclear Medicine, Xiangya Hospital, Central South University, 87 Xiangya Road, Changsha, 410008 Hunan China; 4 Department of Vascular Surgery, The Second Affiliated Hospital of University of South China, Hengyang, China; 5 Key Laboratory of Biological Nanotechnology of National Health Commission, Xiangya Hospital, Central South University, Changsha, Hunan China; 6 National Clinical Research Center for Geriatric Disorders (XIANGYA), Changsha, China

**Keywords:** Prostate cancer, PSMA, PET/CT, MRI, Prostate-specific membrane antigen

## Abstract

**Purpose:**

To assess ^68^Ga-PSMA PET/CT for detection of low- and intermediate-risk prostate cancer (PCa), high-risk PCa in comparison with mpMRI, respectively, and to determine which of low- and intermediate-risk PCa are more likely to be detected by ^68^Ga-PSMA PET/CT.

**Methods:**

We conducted a retrospective analysis of patients who had undergone a prostate biopsy and/or radical prostatectomy and who were scanned with ^68^Ga-PSMA PET/CT and mpMRI between June 2019 and March 2021. The mpMRI images were scored with the Prostate Imaging-Reporting and Data System Version 2.1 (PI-RADS) and were classified as either negative (PI-RADS 1–3) or positive (PI-RADS 4–5). Suspicious ^68^Ga-PSMA PET/CT lesions were reviewed for each relevant patient and classified by double-trained board-certified nuclear medicine physicians. The results were evaluated with the histopathological outcome. All patients were classified according to the D’Amico classification, and the clinical data were combined for stratified analysis.

**Result:**

A total of 101 patients who were pathologically diagnosed with PCa were analyzed. Of the 101 patients, 88 (80.6%) patients presented with a pathologic mpMRI, and 85 (79.1%) with a pathologic ^68^Ga-PSMA PET/CT. In the high-risk PCa cohort, ^68^Ga-PSMA PET/CT was positive in 64/66 (97.0%) patients and yielded a higher detection rate than that for the mpMRI patients (58/66, 87.9%; *p* < 0.05). However, mpMRI provided superior diagnostic confidence in identifying low- and intermediate-risk PCa (30/35, 85.7% vs. 21/35, 60.0%; *p* < 0.05). When the age threshold exceeded 62.5 years and the serum prostate specific antigen (PSA) threshold exceeded 9.4 ng/ml, a higher uptake of PSMA was more likely to occur in the lesions of low- and intermediate-risk PCa.

**Conclusion:**

The diagnostic performance of ^68^Ga-PSMA PET/CT was superior to that of mpMRI in the high-risk PCa cohort, which was consistent with prior studies. Furthermore, in the initial diagnosis of low- and intermediate-risk PCa, we found that mpMRI showed a higher diagnostic accuracy than ^68^Ga-PSMA PET/CT did. Low- and intermediate-risk PCa patients with a PSA ≥ 9.4 ng/ml and age ≥ 62.5 years were more likely to have a positive ^68^Ga-PSMA PET/CT result.

**Supplementary Information:**

The online version contains supplementary material available at 10.1186/s13550-022-00881-3.

## Introduction

Prostate cancer (PCa) remains one of the most prevalent cancers in the world and a significant cause of death in many regions [1]. The natural course of PCa starts as a disease localized to the prostate, which is followed by a noncastrate rising prostate-specific antigen (PSA) [2]. Different stages of PCa directly affect both the therapeutic schedule and patient prognosis. For example, nonmetastatic PCa includes active surveillance, radical prostatectomy, radiotherapy, and its derivatives [3]. Therefore, the early detection of PCa is of great importance for proper disease management and is dependent on the accuracy of imaging to quantify the extent and location of lesions [4, 5].

Prostate multiparametric magnetic resonance imaging (mpMRI) is a readily available and well-established technique for the evaluation of PCa, which has favorable diagnostic accuracy in PCa detection, and can decrease unnecessary biopsies and instances of nonsignificant PCa diagnoses [6, 7]. mpMRI is evaluated using the Prostate Imaging-Reporting and Data System (PI-RADS) and is currently revolutionizing the PCa diagnostic pathway [8]. However, although mpMRI has a high specificity, it has a poor and heterogeneous sensitivity for local PCa staging, which is important for clinical decision-making and patient counseling [9, 10].

Gallium-68 prostate-specific membrane antigen positron emission tomography/computer tomography (^68^Ga-PSMA PET/CT) is a relatively new nuclear imaging modality, showing high sensitivity and specificity [11]. Recently, several studies have investigated the role of ^68^Ga-PSMA PET/CT in a first-line diagnostic setting and especially in patients with high-risk and biochemically recurrent PCa [11, 12]. The impact of ^68^Ga-PSMA PET/CT on decision-making and disease management has been reviewed, and it is clear that ^68^Ga-PSMA PET/CT has significantly impacted clinical decision-making, especially for the high-risk PCa cohort [13]. However, some studies have also investigated the limitation of ^68^Ga-PSMA PET in detecting low- and intermediate-risk PCa, which is due to the low prevalence of extraprostatic disease, especially to low levels of PSA [14, 15]. Thus far, few studies have provided a direct comparison between mpMRI and ^68^Ga-PSMA PET/CT for the detection of low- and intermediate-risk primary PCa. Consequently, no guidelines advising which modality is preferable for diagnosing the specific level of PCa have been available until recently. We attempted to compare the diagnostic performance of ^68^Ga-PSMA PET/CT with that of mpMRI for the detection of low- and intermediate-risk PCa cohort, high-risk PCa cohort, respectively, and further compared these modalities to histopathology. The secondary goal was to evaluate the additional value of ^68^Ga-PSMA PET/CT for the detection or staging of low- and intermediate-risk PCa.

## Material and methods

### Patients

This retrospective, single-institution study enrolled 101 consecutive patients who underwent 3.0 Tesla (T) mpMRI and ^68^Ga-PSMA PET/CT for suspicious PCa from June 2019 to March 2021. Each of these patients also subsequently underwent a transrectal ultrasound (TRUS)/magnetic resonance imaging (MRI) fusion-guided biopsy, or/and radical prostatectomy (RP). Individuals were excluded from the study based on the following exclusion criteria: (a) Gleason score (GS) or mpMRI outcome not available; and (b) recent prior initiation of systemic treatment such as androgen deprivation therapy (ADT), chemotherapy, or radiotherapy.

Patients were categorized into 3 different risk groups (low risk, intermediate risk, and high risk), according to their clinical primary tumor stage, serum PSA levels, and GS (D’Amico classification, Additional file 1: Table S1) [16]. The study protocol was approved by the Ethics Committee of the Xiangya Hospital Central South University, and written informed consent was obtained from all included patients.

### mpMRI examination and image evaluation

For patients undergoing mpMRI in our hospital, a 3.0 T MR scanner with a specific imaging protocol for the prostate was used. T1-weighted (T1w) axial VIBE sequences (2 mm ST) and T2-weighted (T2w) imaging in three planes were included. During contrast injection, T1w VIBE perfusion imaging was performed. Two separately performed sequences with diffusion-weighted imaging (DWI; b values 1500) and apparent diffusion coefficient (ADC) focused on the whole pelvis and prostatic fossa.

All scans were reviewed and interpreted by 2 radiologists (at least 5 years of prostate mpMRI experience) who were blinded to the PET/CT results. Regions of interest (ROIs) were defined as regions with an abnormal signal on the mpMRI and were contoured and scored with PI-RADS version 2.1 (Additional file 1: Table S2, Additional file 1: Table S3) [17]. Lesions were given a category score from 1 to 5, both experts reviewed discordant results to reach a consensus. Lesions with a score between 1 and 3 were considered negative results, while lesions with a score of 4 or 5 were considered highly likely to be PCa. All patients underwent mpMRI of the prostate within 3 months of PET/CT imaging.

### ^68^Ga-PSMA PET/CT examination and image evaluation

The production process of ^68^Ga-PSMA has been previously described [18]. One hour before scanning, all patients were given an intravenous injection of ^68^Ga-PSMA. The acquisition parameters of the PET/CT were described in previous literature [19]. PET/CT imaging was independently evaluated and reported the presence of suspicious lesions by two double-trained board-certified nuclear medicine physicians who were blinded to the mpMRI and pathological results. Maximum standardized uptake values (SUVmax) of all suspicious lesions and prostate gland background for negative patients were measured. A receiver operating characteristic (ROC) curve analysis has been performed to determine the threshold value of SUVmax to discriminate positive or negative of suspicious ^68^Ga-PSMA PET/CT lesions, and the cutoff calculated by the Youden-selected threshold for SUVmax was 7.9 (Area under the curve (AUC) = 0.995; 95% confidence interval (CI): 0.986–1.000; *p* < 0.001) (Additional file 1: Table S4, Additional file 1: Fig. S1). In case of disagreement, mutual re-evaluation of images was performed to achieve a consensus.

### Histopathology examination and lesion concordance

All patients underwent a TRUS/MRI fusion-guided biopsy or/and robotic-assisted laparoscopic RP and lymph node (LN) dissection. All suspicious lesions in MRI were fused and targeted in real time with the TRUS images, which permit transperineal prostate biopsy with rigid fusion in MRI transrectal ultrasound fusion prostate biopsy. There were no more than two most suspicious lesions were labeled in mpMRI and each suspicious lesions underwent targeted biopsy with 2–4 cores. We also used a 12-core transperineal systematic biopsy for each patient [20]. Histopathological analysis and reporting were performed by experienced uropathologists according to the International Society of Urological Pathology (ISUP) standard protocols and structured according to the 2014 ISUP Gleason grading guidelines [21]. For concordance analysis, both whole-gland RP histology and prostate biopsy histology were analyzed. To compare lesions with biopsy histology, biopsy location was described by the operating surgeon and was correlated with both operation and pathology reports [22]. All lesions described within the imaging reports for ^68^Ga-PSMA PET/CT and mpMRI were considered, as previously described [22].

Because clinical and ethical standards for patient management did not allow surgery or sampling of all detected metastatic lesions, follow-up imaging (^68^Ga-PSMA PET/CT, MRI, CT, scintigraphy) and clinical follow-up findings were used as a modified reference standard to confirm those metastatic lesions that cannot be confirmed by histopathology. A decrease in PSA level, lesion size and/or SUVmax under therapy was regarded as a sign of malignancy. Also, lesions with an increase in size and those with constant or increasing PET positivity were considered malignant [23].

### Statistical analysis

All analyses were performed using SPSS version 26.0 software. Descriptive statistical methods were used to characterize the patient cohort. Patients were categorized into 2 groups: low and intermediate risk, and high risk. Two-sided McNemar’s test was used to analyze and compare the accuracy of the 2 imaging modalities in each group [24]. In the low- and intermediate-risk PCa groups, a *t* test was used to compare the differences of related clinical indicators between positive and negative ^68^Ga-PSMA PET/CT patients. A ROC curve was then analyzed to determine the optimal critical value of meaningful clinical indicators. *p* value of < 0.05 was considered statistically significant.

## Results

### Patient characteristics

**Fig. 1:**
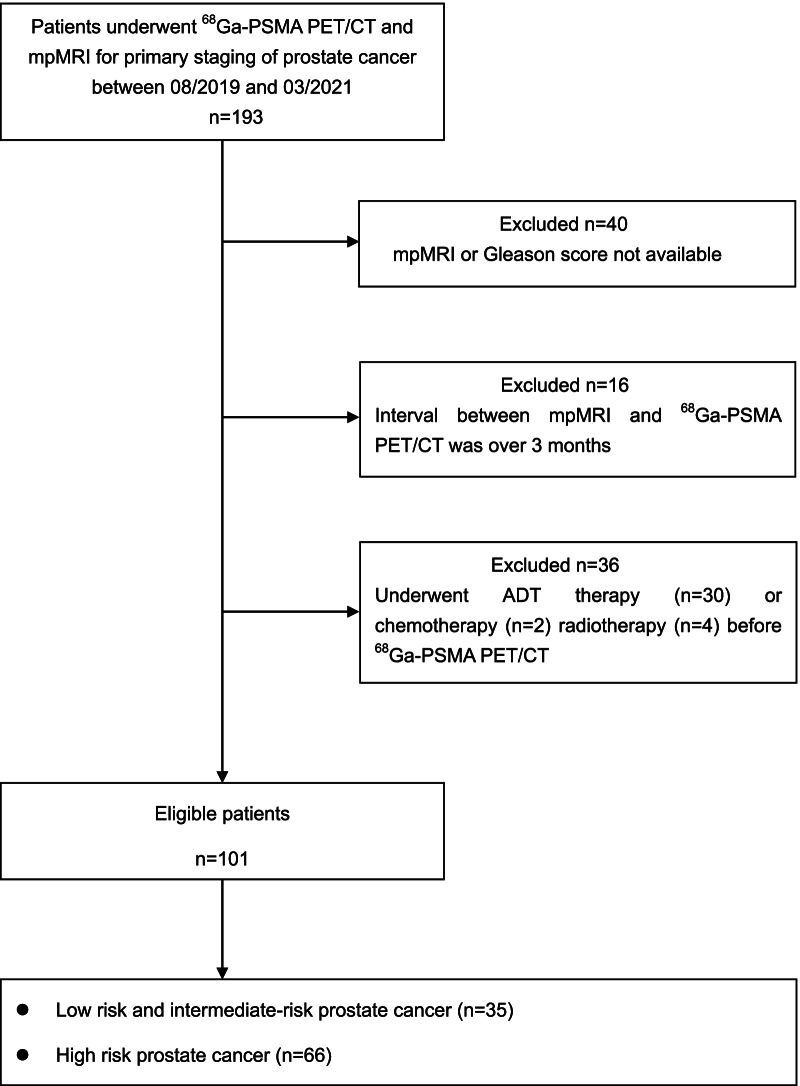
Study flowchart with included and excluded patients as well as the reasons for exclusion. ADT, androgen deprivation therapy. ^68^Ga-PSMA PET/CT, gallium-68 prostate-specific membrane antigen positron emission tomography/computed tomography; mpMRI, multiparametric magnetic resonance imaging

A total of 193 patients newly diagnosed with PCa underwent ^68^Ga-PSMA PET/CT imaging in our institution for primary staging. All patients had an mpMRI of the prostate before the PET/CT scanning. Patients were excluded from the study if their GS or mpMRI results were not available (*n* = 40); the interval between the PET/CT and mpMRI was over 3 months (*n* = 16); or because they underwent treatments such as ADT (*n* = 30), chemotherapy (*n* = 2), and radiotherapy (*n* = 4) prior to PET/CT and MRI. In total, 101 patients were subsequently included in our research (Fig. 1).

The mean age of this group was 68.1 years (range 50–89), and the mean PSA level was 38.1 ng/ml (range 3.5–100.0 ng/ml). GS of the tumors varied between 3 + 3 and 5 + 5 (3 + 3: *n* = 14; 3 + 4: *n* = 12; 4 + 3: *n* = 15; 8: *n* = 28; 9 and 10: *n* = 32). The median time interval between mpMRI and ^68^Ga-PSMA PET/CT in days was 6.0 (IQR: 1.0–23.5). According to the D’Amico classification, low-risk PCa was present in 9/101 (8.9%) patients, intermediate-risk in 26/101 (25.7%), and high-risk in 66/101 (65.4%). Overall, 35 individuals were included in the low- and intermediate-risk PCa groups (Table 1).

**Table 1:** Characteristics of the included prostate cancer patient population (*n* = 101)

Characteristics	Value
Number of patients, *n* (%)	101 (100%)
D’Amico risk classification	
High-risk	66 (65.4%)
Intermediate-risk	26 (25.7%)
Low-risk	9 (8.9%)
Mean age in years	68.1 (50–89)
Median time interval between mpMRI and ^68^Ga-PSMA PET/CT in days	6.0 (1.0–23.5)
Mean PSA in ng/ml	38.1 (3.5–100.0)
ISUP Grade 1 (Gleason score 3 + 3 = 6)	14 (13.9%)
ISUP Grade 2 (Gleason score 3 + 4 = 7)	12 (11.9%)
ISUP Grade 3 (Gleason score 4 + 3 = 7)	15 (14.8%)
ISUP Grade 4 (Gleason score 8)	28 (27.7%)
ISUP Grade 5 (Gleason score 9 and 10)	32 (31.7%)
mpMRI assessment, *n* (%)	
PI-RADS 1–2	0 (0%)
PI-RADS 3	13 (12.9%)
PI-RADS 4	34 (33.7%)
PI-RADS 5	54 (53.4%)

^68^Ga-PSMA PET/CT, gallium-68 prostate-specific membrane antigen positron emission tomography/computed tomography

*ISUP* International Society of Urological Pathology, *mpMRI* multiparametric magnetic resonance imaging, *PI-RADS* prostate imaging-reporting and data system, *PSA* prostate-specific antigen

### High-risk PCa

In total, 66 patients were classified as high-risk PCa, and the demographics of these patients are described in Table 2. The mean age of this group was 69.0 years (range 55–89), and the mean PSA level was 52.3 ng/ml (range 6.1–100.0 ng/ml). GS of the tumors varied between 4 + 3 and 5 + 5 (4 + 3: *n* = 6; 8: *n* = 28; 9 and 10: *n* = 32).

**Table 2:** Characteristics of the high-risk prostate cancer patient population (*n* = 66)

Characteristics	Value
Number of patients, *n* (%)	66 (100%)
Mean age in years	69.0 (55–89)
Median time interval between mpMRI and ^68^Ga-PSMA PET/CT in days	5.0 (0.75–24.0)
Mean PSA in ng/ml	52.3 (6.1–100.0)
ISUP Grade 1 (Gleason score 3 + 3 = 6)	0 (0%)
ISUP Grade 2 (Gleason score 3 + 4 = 7)	0 (0%)
ISUP Grade 3 (Gleason score 4 + 3 = 7)	6 (9.1%)
ISUP Grade 4 (Gleason score 8)	28 (42.4%)
ISUP Grade 5 (Gleason score 9 and 10)	32 (48.5%)
mpMRI assessment, *n* (%)	
PI-RADS 1–2	0 (0%)
PI-RADS 3	8 (12.1%)
PI-RADS 4	14 (21.2%)
PI-RADS 5	44 (66.7%)

^68^Ga-PSMA PET/CT, gallium-68 prostate-specific membrane antigen positron emission tomography/computed tomography

*ISUP* International Society of Urological Pathology, *mpMRI* multiparametric magnetic resonance imaging, *PI-RADS* prostate imaging-reporting and data system, *PSA* prostate-specific antigen

**Fig. 2:**
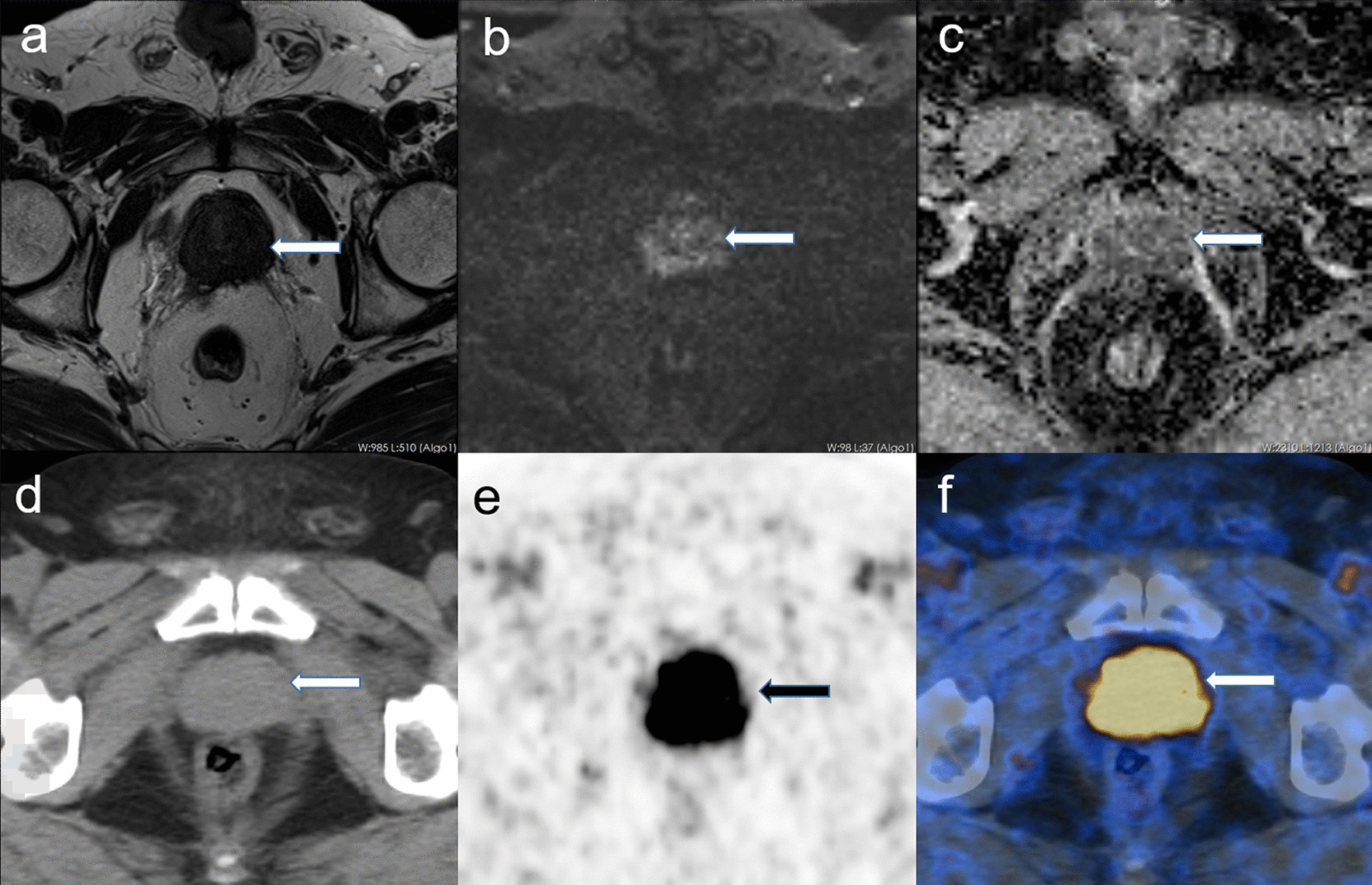
High-risk PCa with mpMRI ( −) and ^68^Ga-PSMA PET/CT (+). A 69-year-old patient whose serum PSA level was 15.4 ng/ml and whose GS was 5 + 5. There is no significant lesion in the prostate gland in the pelvic MRI scan (**a** T2w; **b** b 1500 DWI), while in the ^68^Ga-PSMA PET/CT there is a strong tracer uptake highly likely to be diagnosed (arrow in **e** and **f**). **a**–**c** MRI; **d**–**f** ^68^Ga-PSMA PET/CT; **a** T2w; **b** b 1500 DWI; **c** ADC map; **d** low-dose CT; **e** maximum intensity projection of the PET; **f** fusion of ^68^Ga-PSMA PET and low-dose CT

We noted that 58 patients had both positive mpMRI and ^68^Ga-PSMA PET/CT result, while only 2 patients (3%) had negative mpMRI and ^68^Ga-PSMA PET/CT result. None of the patients diagnosed as positive from the mpMRI returned a negative ^68^Ga-PSMA PET/CT result. However, 6 individuals who had a negative mpMRI result returned a positive ^68^Ga-PSMA PET/CT result (Fig. 2), 5 of the 6 patients were PI-RADS 3 and 1 was PI-RADS 2. The median SUVmax value of ^68^Ga-PSMA PET of these 6 patients was 12.6 (range 9.0–17.5). When comparing the diagnostic accuracy of the 2 imaging modalities for the high-risk PCa group, we found that the diagnostic performance of ^68^Ga-PSMA PET/CT was superior to that of mpMRI (*p* < 0 0.05) ( Table 3).

**Table 3:** Diagnostic results of mpMRI and ^68^Ga-PSMA PET/CT on high-risk prostate cancer patients

	^68^Ga-PSMA PET/CT		
	Positive	Negative	Total
mpMRI			
Positive	58	0	58 (87.9%)
Negative	6	2	8 (12.1%)
Total	64 (97.0%)	2 (3.0%)	66 (100%)
Two-sided McNemar test			*p* = 0.031

^68^Ga-PSMA PET/CT, gallium-68 prostate-specific membrane antigen positron emission tomography/computed tomography; mpMRI, multiparametric magnetic resonance imaging

For PSMA-avid lymph and distant lesions, 34 patients (51.5%) had suspicion of pelvic LN metastases. The amount and size of the pelvic LNs with a PSMA high uptake ranged from 1 to 10, and from 4 to 65 mm, respectively, with a SUVmax ranging from 2.8 to 68.5 (median 14.6). A further 21 patients (31.8%) had suspicion for bone metastases through ^68^Ga-PSMA PET/CT, with the number of bone metastases ranging from 1 to 13, with a median SUVmax of 12.5 (range: 3.4–60.3). 17 had both bone and LN metastases, while 17 had only LN metastases, and 4 solely bone metastases, although this was not histologically confirmed.

### Low- and intermediate-risk PCa

In all, 35 patients were categorized as low- and intermediate-risk PCa, and the demographics of these individuals are described in Table 4. The mean age was 66.5 years (range 50–76), and the mean PSA level was 11.2 ng/ml (range 3.5–19.0 ng/ml). GS of the tumors varied between 3 + 3 and 4 + 3 (3 + 3: *n* = 14; 3 + 4: *n* = 12; 4 + 3: *n* = 9).

**Table 4:** Characteristics of the low-risk and intermediate-risk prostate cancer patient population (*n* = 35)

Characteristics	Value
Number of patients, *n* (%)	35 (100%)
D’Amico risk classification	
Intermediate-risk	26 (74.3%)
Low-risk	9 (25.7%)
Mean age in years	66.5 (50–76)
Median time interval between mpMRI and ^68^Ga-PSMA PET/CT in days	7.0 (1.0–23.0)
Mean PSA in ng/ml	11.2 (3.5–19.0)
ISUP Grade 1 (Gleason score 3 + 3 = 6)	14 (40.0%)
ISUP Grade 2 (Gleason score 3 + 4 = 7)	12 (34.3%)
ISUP Grade 3 (Gleason score 4 + 3 = 7)	9 (25.7%)
MpMRI assessment, *n* (%)	
PI-RADS 1–2	0 (0%)
PI-RADS 3	5 (14.3%)
PI-RADS 4	20 (57.1%)
PI-RADS 5	10 (28.6%)

^68^Ga-PSMA PET/CT, gallium-68 prostate-specific membrane antigen positron emission tomography/computed tomography

*ISUP* International Society of Urological Pathology, *mpMRI* multiparametric magnetic resonance imaging, *PI-RADS* prostate imaging-reporting and data system, *PSA* prostate-specific antigen

**Fig. 3:**
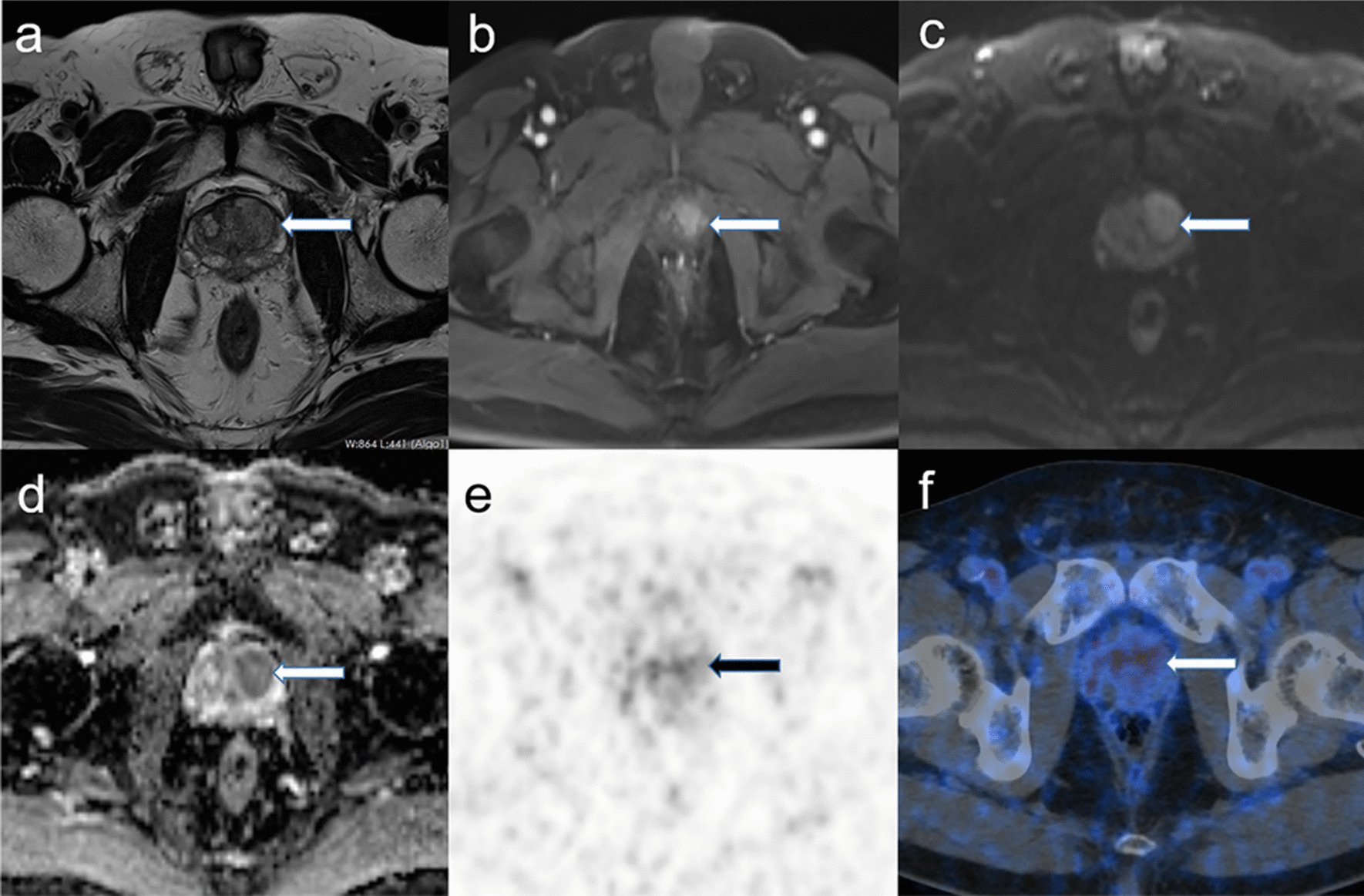
Example of an inconsistent evaluation using mpMRI (+) and ^68^Ga-PSMA PET/CT (−) in low- and intermediate-risk PCa. A 59-year-old patient whose serum PSA level was 8.0 ng/ml, and GS was 3 + 3. There is a lesion in the left transition zone of the prostate gland in the pelvic MRI scan (**a** T2w, arrow; **b** contrast-enhanced T1w sequence, arrow), and a diffusion restriction (**c** b 1500 DWI, arrow; **d** corresponding ADC map, arrow) makes the presence of a large PCa very likely. In the ^68^Ga-PSMA PET/CT there is no strong tracer uptake highly likely to not be diagnosed (arrow in **e** and **f**). **a**–**d** MRI; **e**–**f** ^68^Ga-PSMA PET/CT; **a** T2w; **b**: contrast-enhanced T1w; **c** b 1500 DWI; **d** ADC map; **e** maximum intensity projection of the PET; **f** fusion of ^68^Ga-PSMA PET and low-dose CT

**Fig. 4:**
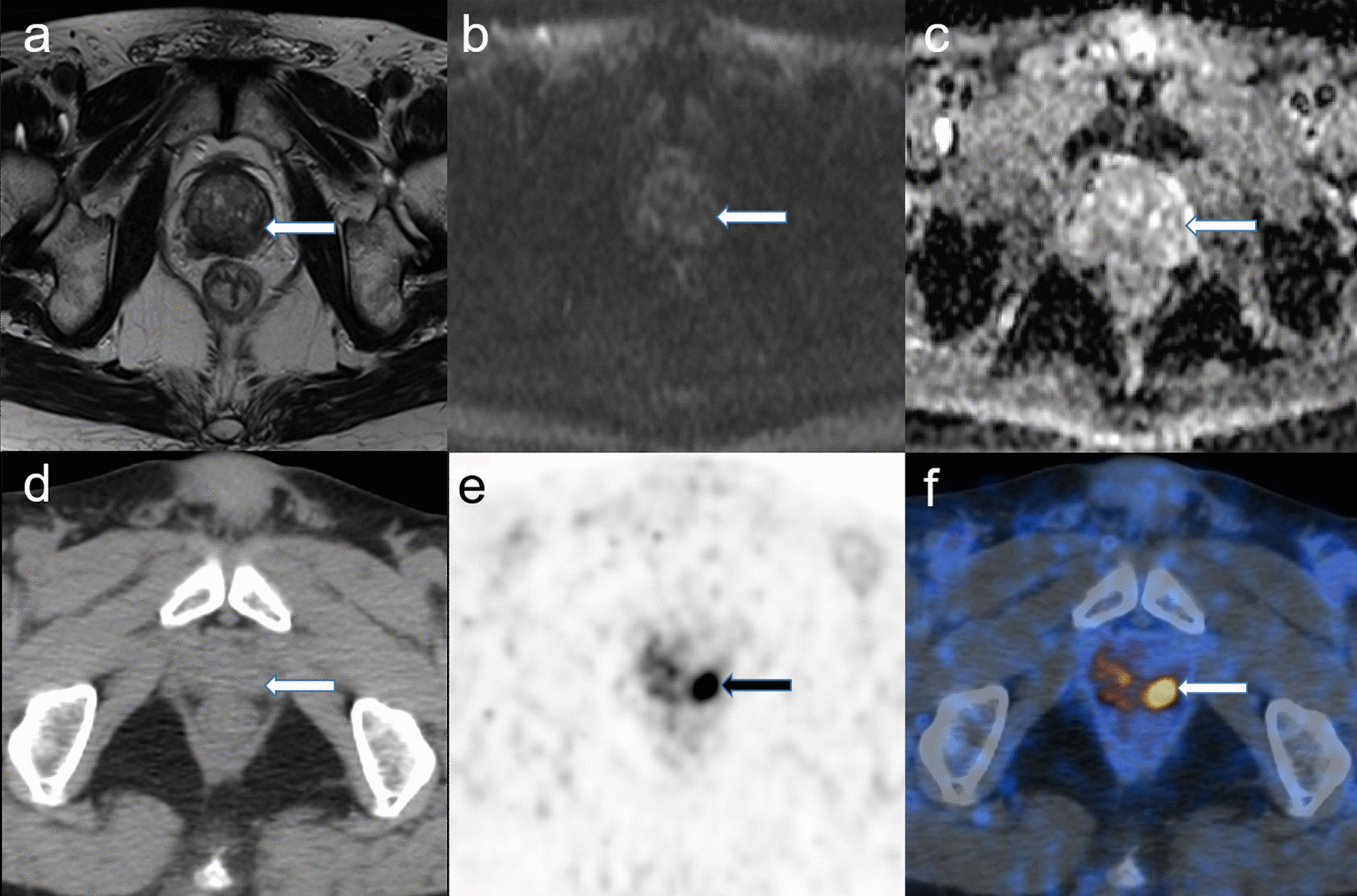
Low- and intermediate-risk PCa with PSA > 9.4 ng/ml and age > 62.5 years, who are mpMRI (−) and ^68^Ga-PSMA PET/CT (+). A 73-year-old patient whose serum PSA level was 17.9 ng/ml and whose GS was 3 + 4. There is no lesion in the prostate gland in the pelvic MRI scan (**a** T2w; **b** b 1500 DWI), while in the ^68^Ga-PSMA PET/CT, there is a strong tracer uptake highly likely to be diagnosed (arrow in **e** and **f**). **a**–**c** MRI; **d**–**f** ^68^Ga-PSMA PET/CT; **a** T2w; **b** b 1500 DWI; **c** ADC map; **d** low-dose CT; **e** maximum intensity projection of the PET; **f** fusion of ^68^Ga-PSMA PET and low-dose CT

We found that 18 patients had both a positive mpMRI and ^68^Ga-PSMA PET/CT result, while 2 patients had both a negative mpMRI and ^68^Ga-PSMA PET/CT result. Twelve individuals were diagnosed positive through the mpMRI, but returned a negative ^68^Ga-PSMA PET/CT result (Fig. 3), the PI-RADS scores of 8 patients were 4 while 4 patients were PI-RADS 5, and the median SUVmax value of ^68^Ga-PSMA PET of these 12 patients was 5.6 (range 3.7–7.8). Only 3 individuals had a negative mpMRI and positive ^68^Ga-PSMA PET/CT result (Table 5; Fig. 4). Comparison of the 2 imaging modalities was performed by means of a 2-sided McNemar’s test. We found that the diagnostic performance of mpMRI was superior to that of ^68^Ga-PSMA PET/CT for the low- and intermediate-risk PCa group (*p* < 0.05).

**Table 5:** Diagnostic results of mpMRI and ^68^Ga-PSMA PET/CT on all low-risk and intermediate-risk prostate cancer patients

	^68^Ga-PSMA PET/CT		
	Positive	Negative	Total
mpMRI			
Positive	18	12	30 (85.7%)
Negative	3	2	5 (14.3%)
Total	21 (60.0%)	14 (40.0%)	35 (100%)
Two-sided McNemar test			*p* = 0.035

^68^Ga-PSMA PET/CT, gallium-68 prostate-specific membrane antigen positron emission tomography/computed tomography

*mpMRI* multiparametric magnetic resonance imaging

After undergoing ^68^Ga-PSMA PET/CT, 3 patients who were diagnosed with low- and intermediate-risk PCa by traditional indicators had suspicion for pelvic LN metastases, which would upregulate clinical staging of PCa and change the treatment strategy.

**Fig. 5:**
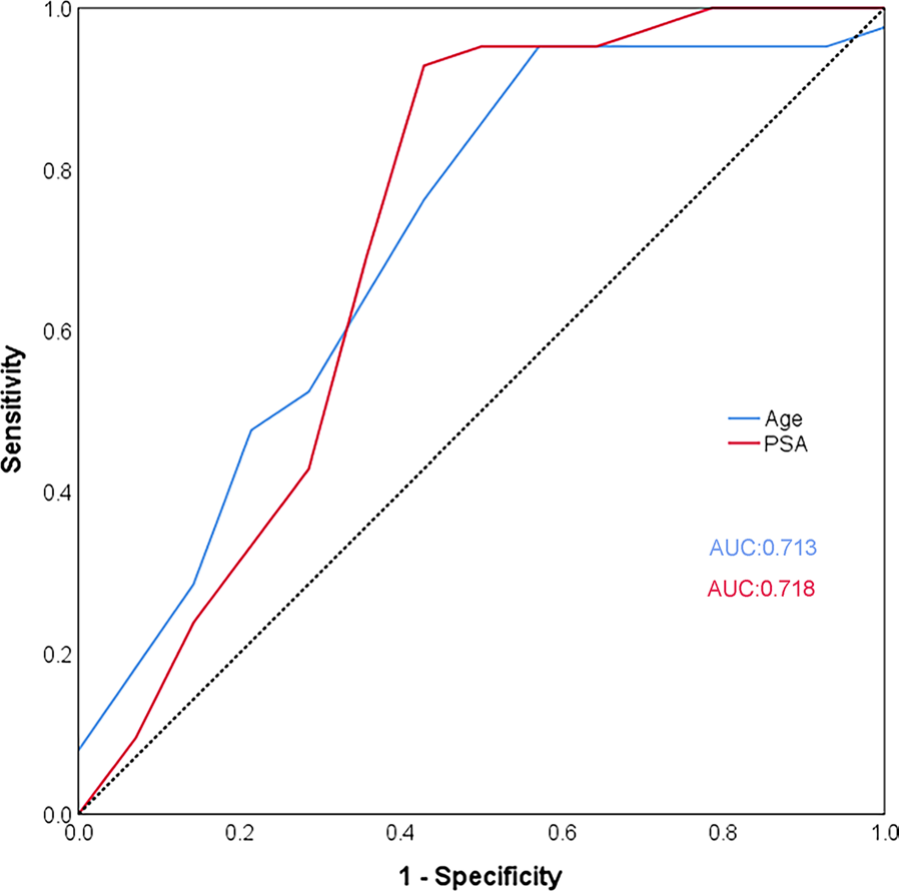
ROC analysis of age and serum PSA of ^68^Ga-PSMA PET/CT for the detection of low- and intermediate-risk PCa

In the low- and immediate risk PCa group, the PSA level was significantly higher in the ^68^Ga-PSMA PET/CT positive patients than in ^68^Ga-PSMA PET/CT negative patients (15.7 ± 6.8 ng/ml vs. 8.8 ± 4.2 ng/ml; *p* = 0.014). The cutoff calculated by the Youden-selected threshold for PSA was 9.4 ng/ml (AUC = 0.718; 95% confidence interval [CI] 0.521–0.915; *p* < 0.05). The age was also significantly higher in the ^68^Ga-PSMA PET/CT positive patients than in the ^68^Ga-PSMA PET/CT negative patients (67.2 ± 10.8 vs. 57.6 ± 8.8; *p* = 0.014). The cutoff calculated by the Youden-selected threshold for age was 62.5 years old (AUC = 0.713; 95% CI 0.532–0.894; *p* < 0.05) (Fig. 5; Table 6). When the age threshold exceeded 62.5 years and the PSA threshold exceeded 9.4 ng/ml, lesion high uptake of PSMA was more likely to occur in the low- and intermediate-risk PCa group. Therefore, for individuals who were above the threshold for the 2 above characteristics, we found that they would benefit more from ^68^Ga-PSMA PET/CT.

**Table 6:** Diagnostic results of age and PSA for ^68^Ga-PSMA PET/CT on all low-risk and intermediate-risk prostate cancer patients

	AUC (95% CI)	*p*	Youden selected threshold
Age	0.713 (0.532–0.894)	0.035	62.5 years
PSA	0.718 (0.521–0.915)	0.031	9.4 ng/ml

*AUC* area under the curve, *CI* confidence interval, *PSA* prostate specific antigen

## Discussion

In this study, we used mpMRI and ^68^Ga-PSMA PET/CT to retrospectively analyze and compare their diagnostic value for determining whether patients had low-, intermediate-, or high-risk PCa. In the high-risk PCa cohort, the diagnostic performance of ^68^Ga-PSMA PET/CT was superior to that of mpMRI, which was consistent with prior studies [11, 14, 15, 24]. However, by further comparing the 2 modalities in the low- and intermediate-risk PCa group, we found that the diagnostic performance of mpMRI was superior to that of ^68^Ga-PSMA PET/CT and that ^68^Ga-PSMA PET/CT may upregulate the staging of some low- and intermediate-risk PCa individuals. Furthermore, through an exploratory multivariate analysis, we found that some patients who had low- or intermediate-risk PCa, whose age threshold exceeded 62.5 years, and/or whose serum PSA threshold exceeded 9.4 ng/ml may be more likely had a high uptake of PSMA. These results highlighted the value of mpMRI in the initial examination of low- and intermediate-risk PCa, and the possible conditions in which PCa patients might benefit from undergoing a combination of both ^68^Ga-PSMA PET/CT and mpMRI. This was particularly the case for patients who had a high suspicion of low- and intermediate-risk PCa, although a negative mpMRI result was determined.

In the last decade, mpMRI has become the leading imaging modality in the primary detection and localization of PCa. Level 1 evidence has recently shown mpMRI to improve clinically significant PCa diagnosis and to decrease unnecessary biopsies and nonsignificant PCa diagnoses [8, 25]. However, mpMRI is limited by both the specificity of its detection and the subjectivity of its diagnosis, with a meta-analysis having showed that the accuracy of mpMRI for detecting clinically significant PCa varied widely between studies (44–87%). PSMA-based imaging modalities such as ^68^Ga-PSMA PET/CT have developed rapidly and have significantly contributed to disease management. In our high-risk PCa cohort, the diagnostic performance of ^68^Ga-PSMA PET/CT was superior to that of mpMRI. Furthermore, the missed diagnosis rate of mpMRI for some LN metastases and bone metastases was relatively high, which can lead to an underestimation of clinically significant PCa. Of the PSMA-avid lymph and distant lesions found in this study’s high-risk PCa group, 34 patients (51.5%) had suspicion for pelvic LN metastases, 21 (31.8%) had suspicion for bone metastases on ^68^Ga-PSMA PET/CT, and 17 individuals had both bone and LN metastases. In addition, studies have shown that ^68^Ga-PSMA PET/CT can lead to management change in up to 52% of the patients depending on the extent of the disease [11, 14, 26, 27].

^68^Ga-PSMA PET/CT is being increasingly recognized as a powerful tool for the detection and assessment of metastatic disease in PCa. However, conventional abdominal imaging and bone scans are still recommended for staging those diagnosed with high-risk PCa. ^68^Ga-PSMA PET/CT remains expensive and is unavailable as a routine tool, especially for low- and intermediate-risk PCa patients. Currently, the available literature on ^68^Ga-PSMA PET/CT is concentrated on primary staging in high-risk PCa and for PCa patients who demonstrate biochemical recurrence after localized treatment. The results of the current study highlight that ^68^Ga-PSMA PET/CT is more accurate than is mpMRI for high-risk PCa. Our results also suggest that high-risk PCa patients who have both a high PSA level and GS score may be strong candidates for ^68^Ga-PSMA PET/CT. This finding is consistent with previous studies [11]. Only 2 (3%) of the 66 high-risk PCa patients in our study returned a negative result through both ^68^Ga-PSMA PET/CT and mpMRI imaging, as the tumor did not show the PSMA tracer uptake and there were abnormal signals on the mpMRI. The reason for these 2 double-negative results, as well as the underlying biology of high-risk PCa, requires further investigation in the future.

Clinically, the proportion of low- and intermediate-risk PCa patients who were recommended to undergo ^68^Ga-PSMA PET/CT were relatively small. In our study, the low- and intermediate-risk PCa patients who underwent ^68^Ga-PSMA PET/CT accounted for only 34.7% (35/101) of the total cohort. Elaborating whole-body staging with ^68^Ga-PSMA PET/CT might be of limited use in low- and intermediate- risk disease, given the low prevalence of metastases and therefore have limited impact on imaging management. Some studies, which largely included unclassified instances of PCa with a high proportion of high-risk PCa, especially in patients who had ^68^Ga-PSMA PET/CT imaging, generally found that ^68^Ga-PSMA PET/CT had a higher detection rate of primary lesions than did mpMRI [13, 15, 22, 27–29]. There have been very few studies which provide a direct comparison between mpMRI and ^68^Ga-PSMA PET/CT for the detection and evaluation of low- and intermediate-risk PCa. According to our results, after an analysis of low- and intermediate-risk PCa as a separate subgroup, the final lesion detection rate of mpMRI was better than that of ^68^Ga-PSMA PET/CT. This is because, as PSA levels decreased, the lesion detection rate of ^68^Ga-PSMA PET/CT also gradually decreased. This relatively low detection rate could be due to the high occurrence of microlesions (even as small as 1 mm) [29, 30]. Another explanation of the lower diagnostic efficacy of ^68^Ga-PSMA PET/CT could be the weaker biochemical affinity of the ligand to the PSMA receptor in low- and intermediate-risk PCa [3, 14, 31]. In this case, a high anatomical MRI resolution is considered to be more advantageous.

In our study, mpMRI failed to detect low- and intermediate-risk PCa in 3 of 35 patients. In the low- and intermediate-risk group, 18 patients had both a positive mpMRI and ^68^Ga-PSMA PET/CT, while only 2 individuals had both a negative mpMRI and ^68^Ga-PSMA PET/CT. In addition, there were 3 patients who had a negative mpMRI and positive ^68^Ga-PSMA PET/CT (Table 5). From this we found that it would be helpful to identify the specific population who might benefit from ^68^Ga-PSMA PET/CT.

To this end, we found that low- and intermediate-risk PCa patients who returned a negative mpMRI would benefit from a combination of both modalities. In this study, 21/35 had a positive ^68^Ga-PSMA PET/CT result, which indicates that the improvement of combined MRI and PET occurred on lesions with low- and intermediate-risk PCa [32]. Guidelines recommend that for low- and intermediate-risk PCa patients, a biopsy may be more appropriate than using mpMRI alone (this depends on other factors, such as high PSA, family history, and age) [17, 33]. Our study showed that an additional ^68^Ga-PSMA PET/CT could be helpful for deciding if these low- and intermediate-risk PCa patients should undergo a prostate biopsy or further management [34]. Another key finding was that low- and intermediate-risk PCa patients with a PSA ≥ 9.4 ng/ml and age ≥ 62.5 years were more likely to have a positive ^68^Ga-PSMA PET/CT result. Based on our study of low- and intermediate-risk PCa, we found patients with both a positive mpMRI and ^68^Ga-PSMA PET/CT indicated the possible necessity for a prostate biopsy [35].

We should also note that our study has limitations, including the relatively small number of men who underwent RP and the retrospective nature of the data collection (which had an inherent bias among the reporters). Nonetheless, the final RP specimen remains the most accurate final arbiter to determine presence or absence of PCa on a per-lesion analysis. Moreover, the nuclear medicine physicians of our study were aware that patients had high-risk PCa, which could have increased the risk for confirmation bias. It would have been helpful to have had low- and intermediate-risk PCa patients in this study. However, it is not yet standard of care in our hospital to perform ^68^Ga-PSMA PET/CT imaging in low-risk patients. Lastly, the evaluation of ^68^Ga-PSMA PET/CT was subjective and did not include other objective features like semi-quantification, lesion shape, or location, which may alone account for the low specificity of ^68^Ga-PSMA PET/CT.

## Conclusions

The diagnostic performance of ^68^Ga-PSMA PET/CT was superior to that of mpMRI in the high-risk PCa cohort, which was consistent with prior studies. Furthermore, we found that when compared to ^68^Ga-PSMA PET/CT, mpMRI showed a higher diagnostic accuracy in patients who were initially diagnosed with low- and intermediate-risk PCa. We determined that low- and intermediate-risk PCa patients with a PSA ≥ 9.4 ng/ml and age ≥ 62.5 years were more likely to have a positive ^68^Ga-PSMA PET/CT result. These results may help decide whether patients with low- and intermediate-risk PCa require a prostate biopsy or further management. Further prospective studies are warranted to confirm our findings.

## Supplementary Information

**Additional file 1.** **Supplemental table 1.** D’Amico classification for prostate cancer. **Supplemental table 2.** PI-RADS assessment of peripheral zone. **Supplemental table 3.** PI-RADS assessment of transition zone. **Supplemental table 4.** Diagnostic result of SUVmax for ^68^Ga-PSMA PET/CT to discriminate positive or negative of prostate cancer. **Supplemental fig. 1.** ROC analysis of SUVmax for ^68^Ga-PSMA PET/CT to discriminate positive or negative of prostate cancer.

## Data Availability

Not applicable.
