# Supplementary material for: Comparison of 68Ga-PSMA PET/CT and multiparametric MRI for the detection of low- and intermediate-risk prostate cancer
Source: EJNMMI Res. 2022 Feb 11;12:10. doi: 10.1186/s13550-022-00881-3 (PMC8837766; doi:10.1186/s13550-022-00881-3)
Supplement: Supplementary file 1 — Additional file 1. Supplemental table 1. D’Amico classification for prostate cancer. Supplemental table 2. PI-RADS assessment of peripheral zone. Supplemental table 3. PI-RADS assessment of transition zone. Supplemental table 4. Diagnostic result of SUVmax for 68Ga-PSMA PET/CT to discriminate positive or negative of prostate cancer. Supplemental fig. 1. ROC analysis of SUVmax for 68Ga-PSMA PET/CT to discriminate positive or negative of prostate cancer. [file 13550_2022_881_MOESM1_ESM.docx]

**Supplemental tables and figures of D’Amico classification, PI-RADS assessment, and SUVmax cutoff value of ^68^Ga-PSMA PET/CT**

**Article title:** Comparison of ^68^Ga-PSMA PET/CT and multiparametric MRI for the detection of low- and intermediate-risk prostate cancer

**Journal name:** *EJNMMI Research*

**Author names:** Chuanchi Zhou^1, 2*^, Yongxiang Tang^3*^, Zhihe Deng^4*^, Jinhui Yang^3^, Ming Zhou^3^, Long Wang^1^, and Shuo Hu^3, 5, 6^

**Affiliations:**

^1^ Department of Urology, The Third XiangYa Hospital, Central South University, Changsha, Hunan, P. R. China.

^2^ Department of Urology, Xiangya Hospital, Central South University, Changsha, Hunan, China.

^3^ Department of Nuclear Medicine, Xiangya Hospital, Central South University, Changsha, Hunan, China.

^4^ Department of Vascular Surgery, The Second Affiliated Hospital of University of South China, Hengyang, China.

^5^ Key Laboratory of Biological Nanotechnology of National Health Commission, Xiangya Hospital, Central South University, Changsha, Hunan, China.

^6^ National Clinical Research Center for Geriatric Disorders (XIANGYA)

**E-mail address of the corresponding author:**

Shuo Hu, [hushuo2018@163.com](mailto:hushuo2018@163.com); Long Wang, [wanglong@csu.edu.cn](mailto:wanglong@csu.edu.cn)

- **Supplemental table 1** **D’Amico classification for prostate cancer**
- **Supplemental table 2** **PI-RADS assessment of peripheral zone**
- **Supplemental table 3** **PI-RADS assessment of transition zone**
- **Supplemental table 4 Diagnostic result of SUVmax for ^68^Ga-PSMA PET/CT to discriminate positive or negative of prostate cancer**
- **Supplemental fig. 1 ROC analysis of SUVmax for ^68^Ga-PSMA PET/CT to discriminate positive or negative of prostate cancer**

**Supplemental table 1 D’Amico classification for prostate cancer**

| **Low-risk** | **Intermediate-risk** | **High-risk** | |
| --- | --- | --- | --- |
| PSA <10 ng/mL  and GS <7 (ISUP grade 1)  and cT1-2a | PSA 10-20 ng/mL  or GS 7 (ISUP grade 2/3)  or cT2b | PSA ˃20 ng/mL  or GS ˃7 (ISUP grade 4/5)  or cT2c | any PSA  any GS (any ISUP grade)  cT3-4 or cN+ |
| **Localised** | | | **Locally advanced** |

GS, gleason score; ISUP, International Society for Urological Pathology; PSA, prostate-specific antigen

**Supplemental table 2 PI-RADS assessment of peripheral zone**

| **DWI** | **T2w** | **DCE** | **PI-RADS** |
| --- | --- | --- | --- |
| **1** | Any * | Any | 1 |
| **2** | Any | Any | 2 |
| **3** | Any | - | 3 |
|  |  | + | 4 |
| **4** | Any | Any | 4 |
| **5** | Any | Any | 5 |

* “Any” indicates 1-5

**PI-RADS assessment of DWI:**

Score 1: No abnormality (i.e., normal) on ADC and high b-value DWI

Score 2: Linear/wedge shaped hypointense on ADC and/or linear/wedge shaped hyperintense on high b-value DWI

Score 3: Focal (discrete and different from the background) hypointense on ADC and/or focal hyperintense on high b-value DWI; may be markedly hypointense on ADC or markedly hyperintense on high b-value DWI, but not both

Score 4: Focal markedly hypointense on ADC and markedly hyperintense on high b-value DWI; <1.5cm in greatest dimension

Score 5: Same as 4 but ≥1.5cm in greatest dimension or definite extraprostatic extension/invasive behavior

**PI-RADS assessment for DCE:**

Negative: No early or contemporaneous enhancement; or diffuse multifocal enhancement not corresponding to a focal finding on T2w and/or DWI or focal enhancement corresponding to a lesion demonstrating features of BPH on T2w (including features of extruded BPH in the peripheral zone)

Positive: Focal; and earlier than or contemporaneously with enhancement of adjacent normal prostatic tissues; and corresponding to suspicious finding on T2w and/or DWI

ADC, apparent diffusion coefficient; BPH, benign prostate hyperplasia; DCE, dynamic contrast enhanced; DWI, diffusion-weighted imaging; PI-RADS, Prostate Imaging-Reporting and Data System; T2w, T2-weighted

**Supplemental table 3** **PI-RADS assessment of** **transition zone**

| **DWI** | **T2w** | **DCE** | **PI-RADS** |
| --- | --- | --- | --- |
| **1** | Any * | Any | 1 |
| **2** | ≤3 | Any | 2 |
|  | ≥4 | Any | 3 |
| **3** | ≤4 | Any | 3 |
|  | 5 | Any | 4 |
| **4** | Any | Any | 4 |
| **5** | Any | Any | 5 |

* “Any” indicates 1-5

**PI‐RADS assessment of DWI:**

Score 1: No abnormality (i.e., normal) on ADC and high b-value DWI

Score 2: Linear/wedge shaped hypointense on ADC and/or linear/wedge shaped hyperintense on high b-value DWI

Score 3: Focal (discrete and different from the background) hypointense on ADC and/or focal hyperintense on high b-value DWI; may be markedly hypointense on ADC or markedly hyperintense on high b-value DWI, but not both

Score 4: Focal markedly hypointense on ADC and markedly hyperintense on high b-value DWI; <1.5cm in greatest dimension

Score 5: Same as 4 but ≥1.5cm in greatest dimension or definite extraprostatic extension/invasive behavior

**PI-RADS assessment of T2w:**

Score 1: Normal appearing transition zone (rare) or a round, completely encapsulated nodule. (“typical nodule”)

Score 2: A mostly encapsulated nodule or a homogeneous circumscribed nodule without encapsulation. (“atypical nodule”) or a homogeneous mildly hypointense area between nodules

Score 3: Heterogeneous signal intensity with obscured margins; including others that do not qualify as 2, 4, or 5

Score 4: Lenticular or non-circumscribed, homogeneous, moderately hypointense, and <1.5 cm in greatest dimension

Score 5: Same as 4, but ≥1.5 cm in greatest dimension or definite extraprostatic extension/invasive behavior

ADC, apparent diffusion coefficient; DCE, dynamic contrast enhanced; DWI, diffusion-weighted imaging; PI-RADS, Prostate Imaging-Reporting and Data System; T2w, T2-weighted

**Supplemental table 4 Diagnostic result of SUVmax for ^68^Ga-PSMA PET/CT to discriminate positive or negative of prostate cancer**

|  | **AUC (95% CI)** | ***p*** | **Youden selected threshold** |
| --- | --- | --- | --- |
| **SUVmax** | 0.995 (0.986-1.000) | ˂0.001 | 7.9 |

AUC, area under the curve; CI, confidence interval; SUVmax, maximum standardized uptake values


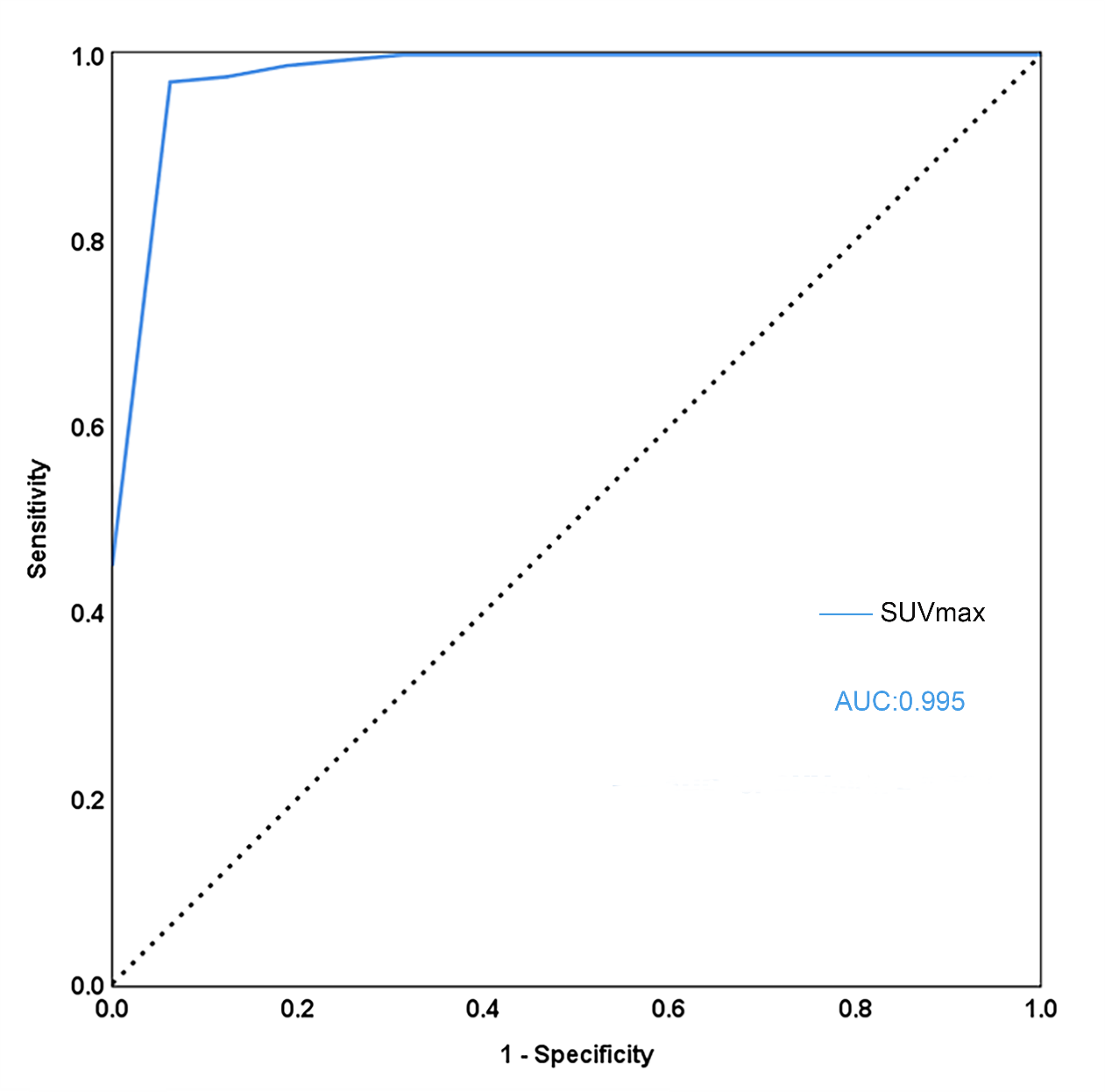


**Supplemental fig. 1 ROC analysis of SUVmax for ^68^Ga-PSMA PET/CT to discriminate positive or negative of prostate cancer**
